# *Aeromonas veronii* biotype sobria serine protease activates aerolysin and cleaves EpCAM to breach the epithelial barrier

**DOI:** 10.1128/jb.00141-26

**Published:** 2026-07-10

**Authors:** Hidetomo Kobayashi, Soshi Seike, Eizo Takahashi, Keinosuke Okamoto, Hiroyasu Yamanaka

**Affiliations:** 1 Laboratory of Molecular Microbiological Science, Faculty of Pharmaceutical Sciences, Hiroshima International University 540030 , Kure, Hiroshima, Japan; 2 Laboratory of Medical Microbiology, Department of Health Pharmacy, Yokohama University of Pharmacy 68348, Yokohama, Kanagawa, Japan; 3 Collaborative Research Center of Okayama University for Infectious Diseases in India, National Institute of Cholera Enteric Diseases 30170, Kolkata, India; University of Virginia School of Medicine, Charlottesville, Virginia, USA

**Keywords:** *Aeromonas veronii *biotype sobria, *Aeromonas *serine protease (ASP), aerolysin, epithelial barrier, tight junction, zonula occludens, EpCAM, claudin-7, transepithelial electrical resistance (TEER)

## Abstract

Infections by *Aeromonas* can rapidly progress from mucosal colonization to invasive disease, but how these bacteria efficiently breach epithelial barriers is unclear. Here, we define a coordinated two-component virulence strategy in *Aeromonas veronii* biotype sobria (*A. veronii* sobria) involving a secreted serine protease (ASP) and aerolysin. In polarized T84 monolayers, wild-type infection caused rapid transepithelial electrical resistance (TEER) loss and degradation of junctional/adhesion proteins. An ASP-deficient strain exhibited delayed barrier collapse with reduced cleavage of ZO-1/2/3 and EpCAM, and purified ASP restored these effects, indicating ASP is required but alone insufficient for full disruption. A transposon screen identified aerolysin and secretion-related genes as key contributors to junctional damage. Mechanistically, ASP directly converted recombinant pro-aerolysin into a hemolytically active toxin *in vitro*, linking protease activity to toxin maturation. Functionally, ASP and aerolysin synergized to promote bacterial translocation across epithelial monolayers with marked sequence dependence; pro-aerolysin pretreatment enhanced ASP-mediated degradation of ZO proteins, claudin-7, and EpCAM. ASP further cleaved EpCAM at multiple extracellular sites, compromising adhesion and junctional homeostasis. Together, these findings establish an ASP–aerolysin pathway that couples toxin activation with junctional proteolysis to drive structural epithelial breaches.

**IMPORTANCE:**

*Aeromonas* infections can rapidly progress from intestinal colonization to invasive disease, yet the molecular steps enabling epithelial breach are unclear. We show that *Aeromonas veronii* sobria uses a coordinated two-factor pathway in which *Aeromonas* serine protease (ASP) both activates the pore-forming toxin aerolysin and directly cleaves key junctional/adhesion proteins, including ZO-1/2/3 and EpCAM. Aerolysin primes cells for enhanced ASP-mediated proteolysis in an order-dependent manner, converting TEER loss into a physical barrier rupture that permits massive bacterial translocation. Targeting ASP–aerolysin cooperation may provide a rational anti-virulence strategy.

## INTRODUCTION

Infections caused by *Aeromonas* species represent an increasingly recognized clinical challenge, with recent epidemiological studies highlighting their growing relevance in human pathogenesis (1–4). *A. veronii* biotype sobria (*A. veronii* sobria), *A. hydrophila*, and *A. dhakensis* cause infections ranging from gastroenteritis and soft tissue infections to life-threatening bacteremia and sepsis (3, 5–7). Recent genomic analyses have revealed substantial diversity in virulence factors among *Aeromonas* species, with *A. dhakensis* emerging as particularly virulent in clinical settings (7, 8). The clinical significance of these pathogens is demonstrated by their ability to cause severe soft tissue infections through the production of extracellular virulence factors and tissue destruction (6, 9–11).

The intestinal epithelial barrier is the first line of defense against enteric pathogens and comprises a complex network of intercellular junctions that maintain tissue integrity while regulating selective permeability (9). Tight junctions formed by transmembrane proteins, including claudins and occludin, are anchored to the cytoskeleton through zonula occludens proteins (ZO-1, ZO-2, and ZO-3) (10, 11). Previous studies have shown that ZO-1 and ZO-2 exhibit functional redundancy in maintaining tight junction organization and epithelial barrier integrity; simultaneous inhibition of ZO-1 and ZO-2 disrupts the membrane localization of multiple tight junction proteins and increases paracellular permeability, supporting their essential roles in epithelial barrier homeostasis (12–14). Although ZO-1 is dispensable for steady-state barrier function, it plays a critical role in mucosal repair processes (15). Claudin-7, a key tight junction component, is essential for intestinal epithelial stem cell self-renewal and differentiation, and its deficiency promotes experimental colitis and associated carcinogenesis (16–20).

The epithelial cell adhesion molecule (EpCAM) has emerged as a crucial regulator of epithelial barrier maintenance and repair (19, 21). Recent studies have demonstrated that EpCAM proteolysis releases complexed claudin-7, which contributes to the repair and maintenance of tight junction barriers (19, 21). The EpCAM‒claudin-7 pathway represents a novel mechanism for epithelial barrier repair, with EpCAM cleavage serving as a regulated process that mobilizes claudin-7 for junctional remodeling (19, 21). The intestinal epithelium forms a highly dynamic and selective barrier, and tight junction claudins are regulated in part through inflammatory mediators to maintain mucosal homeostasis and modulate barrier function during inflammation (22).

Bacterial pathogens have evolved diverse strategies to compromise epithelial barrier integrity, with protease-mediated degradation of junctional proteins being a common virulence mechanism (23–26). Recent studies have highlighted the role of bacterial secreted proteases in disrupting cell-to-cell junctions, as demonstrated by *Helicobacter pylori* HtrA, which specifically targets tight junction components, including occludin and claudin-8 (27). In addition, *Listeria monocytogenes* HtrA has been reported to interact with and proteolyze extracellular matrix molecules, suggesting a potential role in host barrier crossing (28).

*A. veronii* sobria produces two major virulence factors that contribute to pathogenesis: a serine protease (ASP) and a pore-forming aerolysin. ASP belongs to the kexin family of subtilase proteases and exhibits defined cleavage preferences (29, 30). Structural studies have revealed that ASP functions through an external chaperone mechanism, with its carboxy-terminal tail playing a critical role in proper folding and secretion (31, 32). Previous research has demonstrated that ASP can degrade various host proteins and contribute to pathogenesis through multiple mechanisms, including fibrinogen, complement, and prothrombin activation (33–37). However, recent studies using *A. veronii* sobria serine protease have shown that it can degrade several tight junction protein components and facilitate bacterial translocation across epithelial monolayers (38, 39). Both ASP and aerolysin are secreted extracellularly. ASP is synthesized as an immature enzyme; its proper folding and maturation require the external chaperone ORF2, which assists propeptide processing during secretion to yield the active protease (31, 32). Pro-aerolysin is secreted as an inactive precursor via the type II secretion system (T2SS) and requires proteolytic cleavage of its C-terminal domain for activation (40, 41). The precise concentrations of these virulence factors in culture supernatants under the conditions used here have not been directly determined.

*Aeromonas* aerolysin is produced as an inactive precursor (pro-aerolysin) that requires proteolytic activation to generate a mature pore-forming toxin (40). In intestinal epithelial monolayers, aerolysin has been shown to compromise barrier function primarily by reducing paracellular resistance, accompanied by redistribution of sealing tight junction proteins and impaired epithelial wound restitution, supporting a direct role for aerolysin in tight junction dysfunction (42). Recent work further supports the pathological relevance of aerolysin-producing *Aeromonas* in the gut, linking aerolysin to increased susceptibility to colitis *in vivo* (43).

Despite advances in understanding individual virulence factors, significant knowledge gaps remain regarding the potential synergistic interactions between ASP and aerolysin in the disruption of epithelial barriers. While both factors have been implicated in pathogenesis, their cooperative mechanisms and the temporal sequence of their actions have not been systematically investigated. In addition, the specific molecular targets of ASP within the epithelial barrier, particularly the recently identified repair mechanisms involving EpCAM and claudin-7, remain undefined.

The objectives of this study were as follows: (i) to determine whether ASP and aerolysin act cooperatively to disrupt epithelial barrier function; (ii) to identify the molecular mechanisms underlying their potential synergy; (iii) to characterize the specific targeting of junctional and adhesion proteins by ASP; (iv) to investigate the role of ASP in aerolysin maturation; and (v) to develop an integrated model of *Aeromonas*-mediated epithelial barrier disruption. Understanding these mechanisms is crucial for developing targeted therapeutic strategies and providing insights into the complex interplay between bacterial virulence factors and host barrier defense systems.

## RESULTS

### Involvement of ASP in the destruction of the epithelial barrier

**Fig 1:**
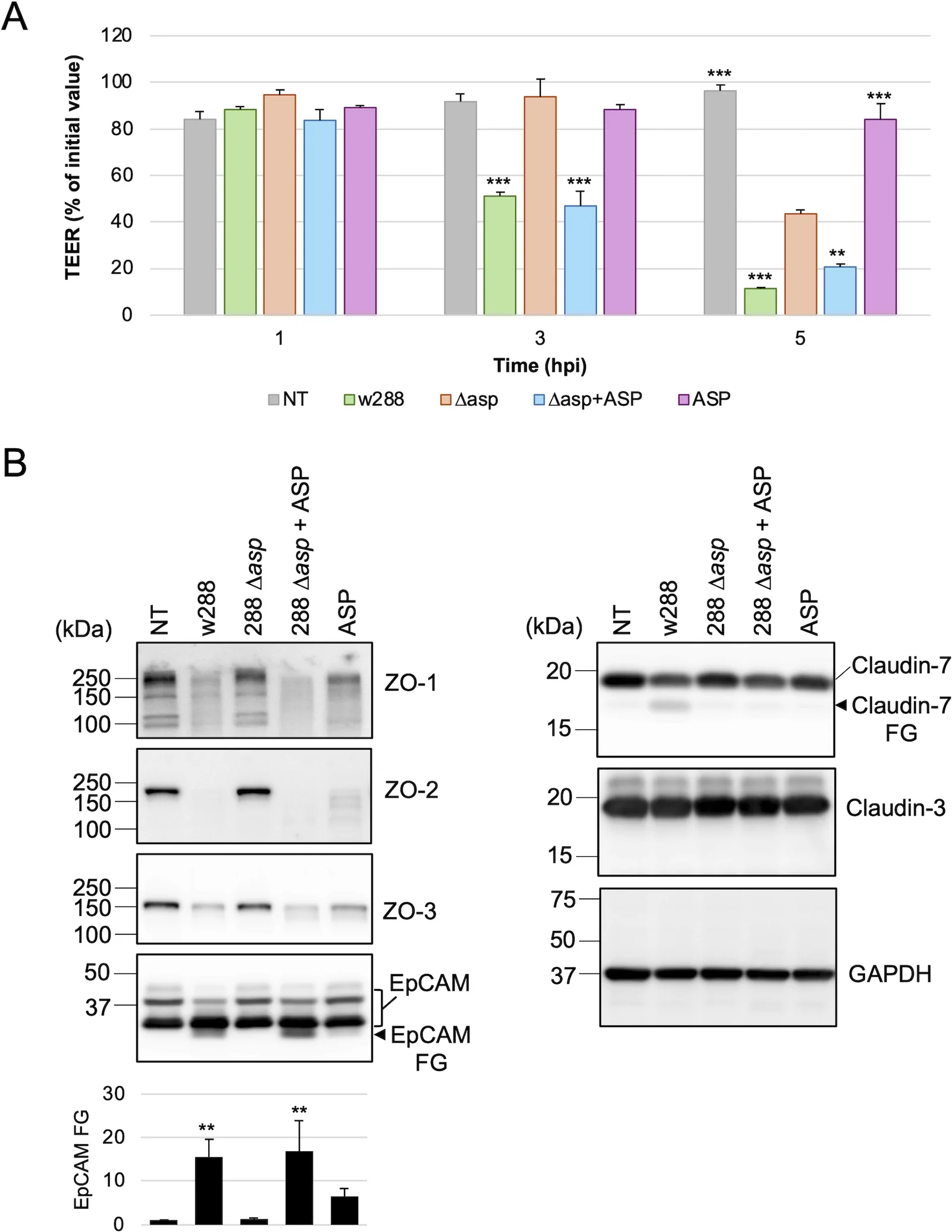
Involvement of ASP in the destruction of the epithelial barrier. (**A**) Time course of transepithelial electrical resistance (TEER) in T84 cells under five conditions: untreated (NT), infected with w288, infected with Δ*asp*, infected with Δ*asp* supplemented with purified ASP (Δasp + ASP), and treated with purified ASP alone (ASP). Bacterial infections were performed at a multiplicity of infection (MOI) of 500. Purified ASP was used at 100 nM. All protein concentrations refer to final concentrations in the culture medium. Data are expressed as initial TEER values and represent mean ± SEM (*n* = 3 independent experiments, each performed with a separate T84 monolayer culture). Statistical analysis was performed using one-way ANOVA, followed by Dunnett’s multiple-comparison test using Δasp as the control. *P < 0.05, **P < 0.01, and ***P < 0.001. (**B**) ASP-mediated degradation of junctional and adhesion-related proteins. Representative immunoblots showing the effects of different treatments on tight junction proteins (ZO-1, ZO-2, ZO-3, claudin-7, claudin-3), epithelial adhesion molecule (EpCAM), and GAPDH (loading control) at 3 h post-infection. Upper panels show immunoblot results for each protein under five treatment conditions. Lower panel shows quantitative densitometric analysis of EpCAM degradation fragments expressed as fold-change relative to NT (set to 1.0). Representative immunoblots are shown. Densitometric analyses represent mean ± SEM (*n* = 3 independent experiments). Statistical analysis was performed using one-way ANOVA, followed by Dunnett’s multiple-comparison test using Δasp as the control. *P < 0.05, **P < 0.01, and ***P < 0.001.

To evaluate the impact of *Aeromonas* serine protease (ASP) on epithelial barrier integrity, TEER was measured under five distinct conditions: untreated (NT), infected with the wild-type *A. veronii* sobria strain 288 (w288), infected with its ASP-deficient strain (Δ*asp*), infected with the Δ*asp* strain supplemented with purified ASP (Δ*asp* + ASP), and exposed to purified ASP alone (ASP). The NT control remained stable throughout the observation period, retaining 96.5 ± 2.5% at 5 h post-infection (hpi) (Fig. 1A). The w288 strain caused rapid TEER decline, dropping to 88.3 ± 1.3% at 1 hpi, 51.2 ± 1.7% at 3 hpi, and 11.5 ± 0.5% at 5 hpi. In contrast, the Δ*asp* strain exhibited delayed TEER decline, maintaining significantly higher values at 5 hpi (43.5 ± 1.5% vs 11.5 ± 0.5; *P <* 0.001), indicating that ASP contributes to rapid barrier disruption.

Supplementation of purified ASP to the Δ*asp* strain fully restored the w288-like TEER decline pattern, reaching 20.5 ± 1.4% at 5 hpi. Notably, purified ASP alone did not reduce TEER, which remained at 84.1 ± 6.7% at 5 hpi, demonstrating that ASP is necessary but insufficient for barrier disruption. These results indicate that ASP, in conjunction with other bacterial factors, is required for the rapid loss of epithelial barrier integrity during infection.

Junctional and adhesion-related proteins were assessed by immunoblotting under the same five conditions after 3 hpi. In the wild-type strain (w288) infection, tight junction-associated proteins (ZO-1, ZO-2, ZO-3, and claudin-7) and the epithelial adhesion-related molecule EpCAM were degraded (Fig. 1B). In the Δ*asp* infection, the degradation of these proteins was significantly reduced compared to that in w288 infection, which was consistent with the preserved epithelial barrier function indicated by TEER. Upon the addition of purified ASP to the Δ*asp* infection, degradation of ZO-1/2/3 and EpCAM was restored to levels approaching those of w288 infection, while claudin-7 exhibited only partial restoration. Importantly, while purified ASP alone induced degradation of ZO-1/2/3 and EpCAM, the extent of degradation was modestly reduced compared to that observed in the Δ*asp* strain supplemented with ASP under infection conditions, suggesting that bacterial factors beyond ASP contribute to the enhanced junctional protein degradation observed during wild-type infection. This molecular alteration by ASP alone was not accompanied by a significant decrease in TEER within the observation period (Fig. 1A).

Densitometric quantification of EpCAM degradation fragments expressed as fold-change relative to untreated controls (NT = 1.0) revealed the following approximate increases in degradation fragment intensity: w288 infection, 15 ± 4-fold (*P* < 0.01); Δ*asp* infection, 1.2 ± 0.4-fold; Δ*asp* infection supplemented with purified ASP, 17 ± 7-fold (*P* < 0.01); and purified ASP alone, 6.4 ± 1.9-fold (*P* < 0.05). We note that the degradation fragments are not fully resolved from the intact EpCAM band, and these values should be interpreted as semi-quantitative estimates. These semi-quantitative data further support that ASP alone is insufficient to achieve the full extent of junctional protein degradation observed during bacterial infection, indicating the involvement of additional bacterial factors that enhance ASP-mediated proteolysis.

### Analysis of transposon mutants

Based on the observation that purified ASP alone produced weaker ZO protein degradation compared to ASP in the context of bacterial infection (Fig. 1B), we hypothesized that additional bacterial factors cooperate with ASP to enhance junctional protein degradation.

**Fig 2:**
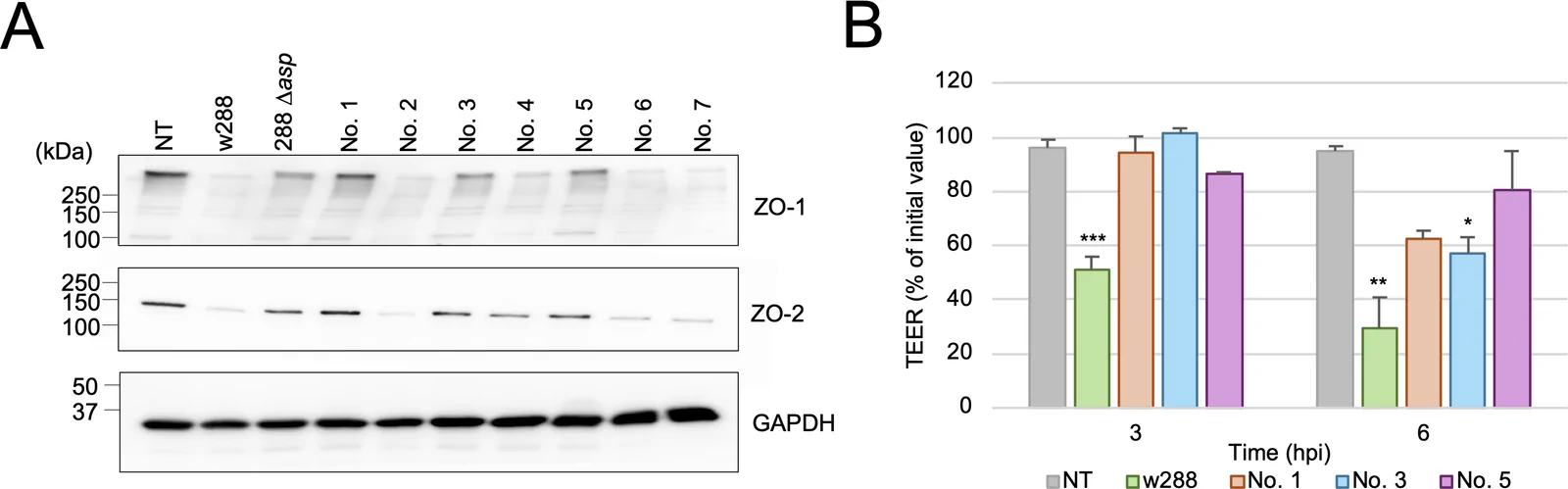
Identification of bacterial factors that contribute to enhanced ZO protein degradation using transposon mutagenesis. (**A**) Transposon mutant screening for ZO protein degradation defects. Immunoblots of ZO-1, ZO-2, and ZO-3 proteins at 3 hpi with w288 or selected transposon mutants from a 5,472-clone library screen. Mutant nos. 1, 3, and 5 showed severely impaired ZO degradation, while No. 4 exhibited moderate impairment. GAPDH, loading control; NT, no treatment. (**B**) Quantitative analysis of epithelial barrier function preservation in transposon mutants. TEER measurements were performed at 0, 3, and 6 h post-infection to evaluate long-term protective effects of transposon mutations on epithelial barrier integrity. Bacterial infections were performed at MOI 500. Data represent mean ± SEM (*n* = 3 independent experiments, each performed with a separate T84 monolayer culture). Statistical analysis was performed using one-way ANOVA, followed by Dunnett’s multiple comparisons test with NT as the control. *P < 0.05, **P < 0.01, and ***P < 0.001.

To identify these factors, a transposon mutant library consisting of 5,472 clones was constructed in w288. The library was first screened by visual assessment of reduced epithelial cell detachment activity under microscopic observation. Seven mutants showing reduced detachment were then subjected to western blot analysis to confirm reduced ZO protein degradation capacity. This screening strategy was designed to identify bacterial genes whose disruption would impair the enhanced junctional protein degradation observed during wild-type infection, thereby revealing factors that normally cooperate with ASP during barrier disruption. The screening identified four mutants with impaired ability to degrade ZO proteins: three mutants (nos. 1, 3, and 5) showed markedly reduced ZO protein degradation, while one mutant (No. 4) exhibited a moderate reduction relative to the wild type (Fig. 2A). The three mutants with severely impaired ZO degradation also showed a suppressed TEER decline compared with w288, linking reduced junctional proteolysis with attenuation of barrier disruption.

To quantitatively assess the impact of the identified mutations on epithelial barrier integrity, TEER measurements were conducted at 0, 3, and 6 hpi for the three mutants showing the most pronounced reduction in ZO protein degradation (Fig. 2B). w288 demonstrated a progressive decline in TEER, dropping to 50.9 ± 4.9% of the initial value at 3 hpi and further decreasing to 29.7 ± 11.4% by 6 hpi. In contrast, all three mutants exhibited significantly attenuated TEER decline compared to the wild type. Mutant No. 1 maintained TEER at 94.6 ± 5.6% (*P <* 0.01); mutant No. 3 preserved TEER at 101.4 ± 2.0% (*P <* 0.001); and mutant No. 5 maintained TEER at 86.6 ± 0.2% (*P <* 0.01). By 6 hpi, mutant No. 5 continued to display significant barrier preservation, retaining 80.8 ± 14.2% of the initial TEER (*P <* 0.05), while mutant nos. 1 (62.2 ± 3.2%) and 3 (56.8 ± 6.1%) showed a trend toward protection that did not reach statistical significance. These quantitative results corroborate the immunoblotting findings, demonstrating that the transposon insertions significantly impair the ability of the bacteria to compromise epithelial barrier function.

Analysis of the transposon insertion sites revealed disruptions in distinct functional genes (Table 1). The insertion in mutant No. 1 was mapped to the aerolysin gene. Mutant No. 3 carried an insertion in a type II secretion system-related gene. Mutant No. 5 harbored an insertion in a processing protease-related gene. In contrast, mutant No. 4, which showed a moderate phenotype, had an insertion in the Lon protease gene, which is involved in protein quality control. To confirm these findings, we assessed ASP protease activity in culture supernatants using an ASP-specific substrate. Both the T2SS-deficient mutant (No. 3) and the prepilin peptidase-deficient mutant (No. 5) completely lacked ASP activity, confirming that ASP secretion requires a functional T2SS machinery (Fig. S3). In contrast, the aerolysin-deficient mutant (No. 1) retained normal ASP activity, indicating that aerolysin itself is not required for ASP secretion. Mutants with intermediate phenotypes (nos. 4, 6, and 7) showed corresponding intermediate levels of ASP activity, suggesting indirect effects on the secretion apparatus or protein stability. The other mutants in the library did not show detectable reductions in junctional protein degradation.

**TABLE 1:** Identification of transposon insertion sites in mutants obtained from the screen for reduced epithelial barrier-disrupting activity^*a*^

Sample no.	Transposon insertion gene	Locus tag
No. 1	Aerolysin	AHA_RS02190
No. 2	Phosphatidylcholine-sterol acyltransferase	AHA_3791
No. 3	Type II secretion system protein L	AHA_RS02895
No. 4	Lon protease	AHA_RS10165
No. 5	Type 4 prepilin-like proteins leader peptide-processing enzyme	AHA_RS19560
No. 6	Ligand-gated channel protein	AHA_RS08370
No. 7	4-Hydroxyphenylpyruvate dioxygenase, flagellar hook-associated protein 3	AHA_RS13450 AHA_RS14315

^*a*^ A mariner-based transposon mutant library of *A. veronii* sobria strain 288 was screened for clones exhibiting reduced epithelial cell detachment and/or reduced degradation of tight-junction scaffold proteins (ZO proteins). The genomic loci disrupted by transposon insertion were determined by sequencing the transposon–genome junction. Gene annotations are shown as the predicted disrupted gene(s) at the insertion site.

**Fig 3:**
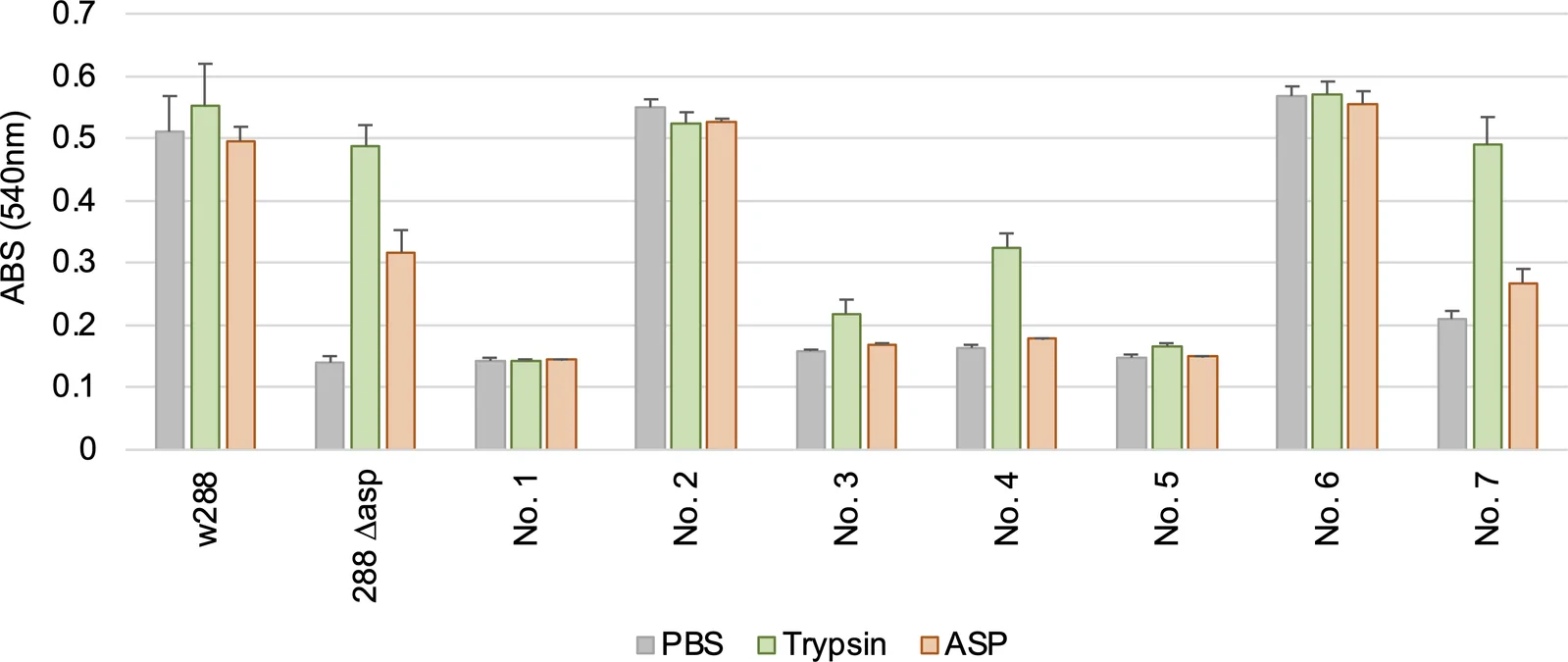
Hemolytic activity of transposon-inserted mutants. Hemolytic activity was assessed by measuring the absorbance at OD540 of supernatants after treatment with 2% sheep red blood cells. Culture supernatants from w288 and transposon mutant strains were mixed with PBS (control), 100 nM trypsin, or 100 nM ASP and incubated at 37˚C for 30 min, then mixed 1:1 with 2% sheep red blood cells and incubated at 37˚C for 1 h before measurement. Data represent mean values from independent experiments.

Since mutant No. 1 carried a transposon insertion in an aerolysin gene, hemolytic activity in culture supernatants was measured to examine the relationship between aerolysin and junctional protein degradation. The three mutants with markedly reduced ZO degradation (nos. 1, 3, and 5) also exhibited substantially lower hemolytic activity than the wild type as shown by the gray bar graphs (Fig. 3).

To test whether the supernatants contained immature aerolysin, an exogenous protease (trypsin or ASP) was added to the supernatants. Hemolytic activity remained at baseline levels in the supernatants from these three mutants, even after protease addition, suggesting an impairment in toxin production and/or secretion rather than extracellular maturation (nos. 1, 3, and 5 in Fig. 3; the green and orange bars). Mutant No. 7 carried insertions in genes encoding 4-hydroxyphenylpyruvate dioxygenase and flagellar hook-associated protein 3 and exhibited a pattern of reduced but not abolished hemolytic activity and intermediate ASP protease activity. The possibility that this or other mutants harbor additional undetected transposon insertions cannot be excluded. In contrast, adding protease to the Δ*asp* strain supernatant yielded high hemolytic activity, consistent with the possibility that extracellular proteolysis can convert pro-aerolysin to the mature toxin outside the cell (288 Δ*asp* in Fig. 3; the green and orange bars).

### Role of ASP in aerolysin maturation

**Fig 4:**
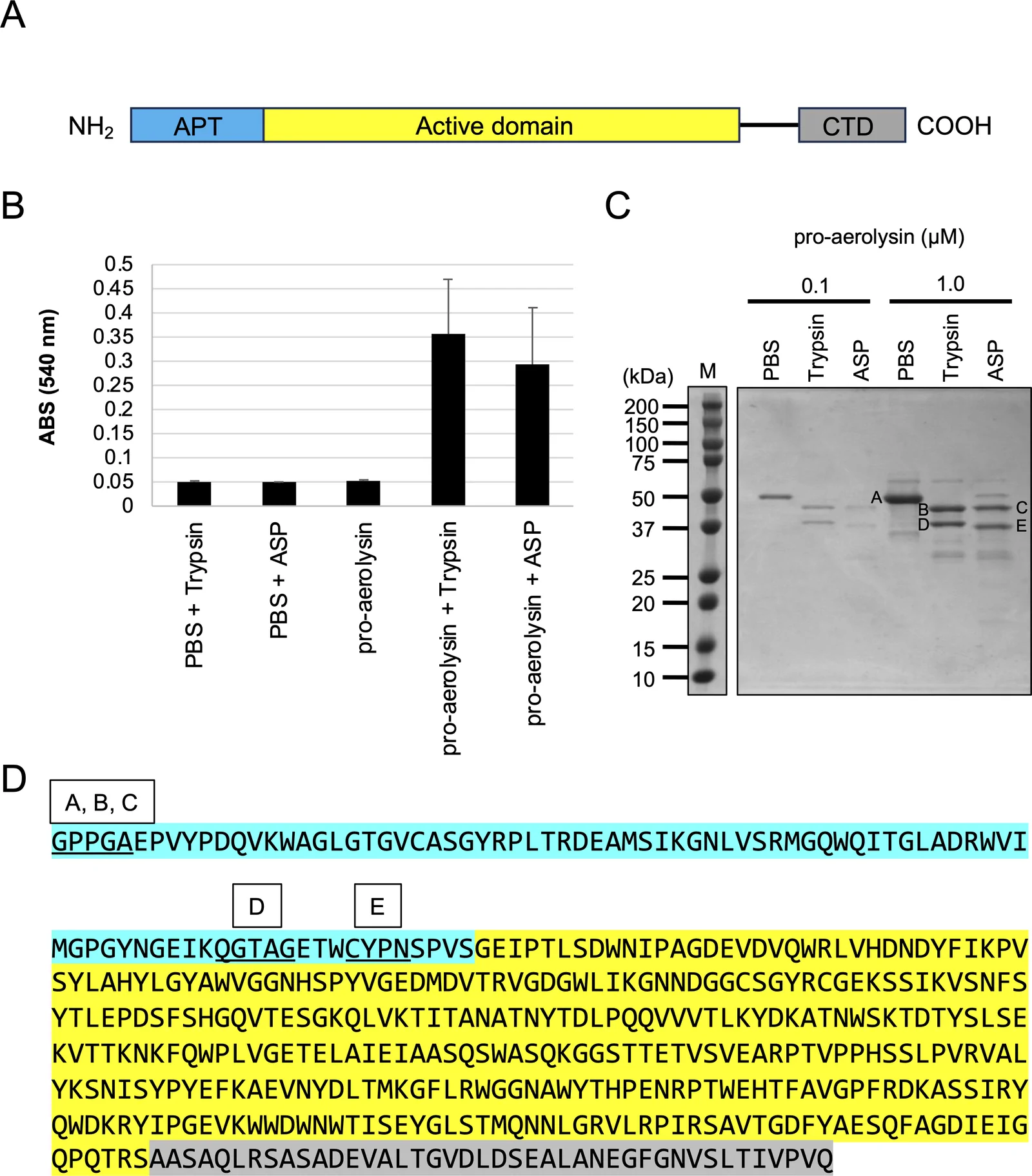
ASP-mediated activation of pro-aerolysin. (**A**) Schematic of pro-aerolysin structure. It is converted to mature aerolysin via C-terminal domain (CTD) cleavage. Aerolysin/pertussis toxin domain (APT) is the N-terminal lectin-like domain of aerolysin that mediates binding to target cells. (**B**) Hemolytic activity (OD540) of pro-aerolysin after treatment with PBS, 100 nM trypsin, or 100 nM ASP at 37˚C for 30 min. (**C**) SDS-PAGE analysis of pro-aerolysin (0.1 and 1.0 µM) treated with PBS, trypsin, or ASP. Protein bands: A (untreated), B and D (trypsin-treated), and C and E (ASP-treated). (**D**) N-terminal sequences of identified bands. Bands B and C show identical N-terminal sequence to pro-aerolysin (GPPGA) but reduced molecular weight, indicating CTD cleavage. Bands D and E show distinct N-terminal sequences (QGTAG and CYPN).

Aerolysin produced by *Aeromonas* forms pores in epithelial membranes, thereby compromising epithelial barrier function. It is produced as a precursor (pro-aerolysin) and converted to a mature toxin via proteolytic C-terminal domain (CTD) cleavage (Fig. 4A) (40).

To examine whether ASP participates in this maturation process, purified pro-aerolysin (100 nM) was incubated with trypsin (100 nM) or ASP (100 nM) at 37˚C for 30 min, followed by hemolytic assays. Hemolytic activity was detected in samples treated with either trypsin or ASP, whereas untreated pro-aerolysin showed no activity (Fig. 4B). SDS-PAGE analysis revealed two distinct fragments after protease treatment (Fig. 4C). N-terminal sequencing indicated that the higher-molecular-weight bands (bands B and C) shared the pro-aerolysin N-terminus (GPPGA), whereas the lower-molecular weight bands (bands D and E) carried the sequences QGTAG and CYPN, respectively (Fig. 4D). The QGTAG N-terminal sequence of band D is consistent with trypsin cleavage at the preceding lysine (K) residue, while the CYPN N-terminal sequence of band E suggests that ASP cleaves at a Trp–Cys (WC) bond within the C-terminal domain. Notably, despite having identical N-terminal sequences to the untreated pro-aerolysin (band A), bands B and C migrated at lower molecular weights, indicating a reduction in molecular weight consistent with C-terminal cleavage. Although cleaved CTD fragments were not directly detected in this analysis, the molecular weight shifts of bands B and C, combined with the acquisition of hemolytic activity, provide indirect evidence of CTD cleavage by both trypsin and ASP.

### Effects of purified ASP and pro-aerolysin on epithelial barrier

**Fig 5:**
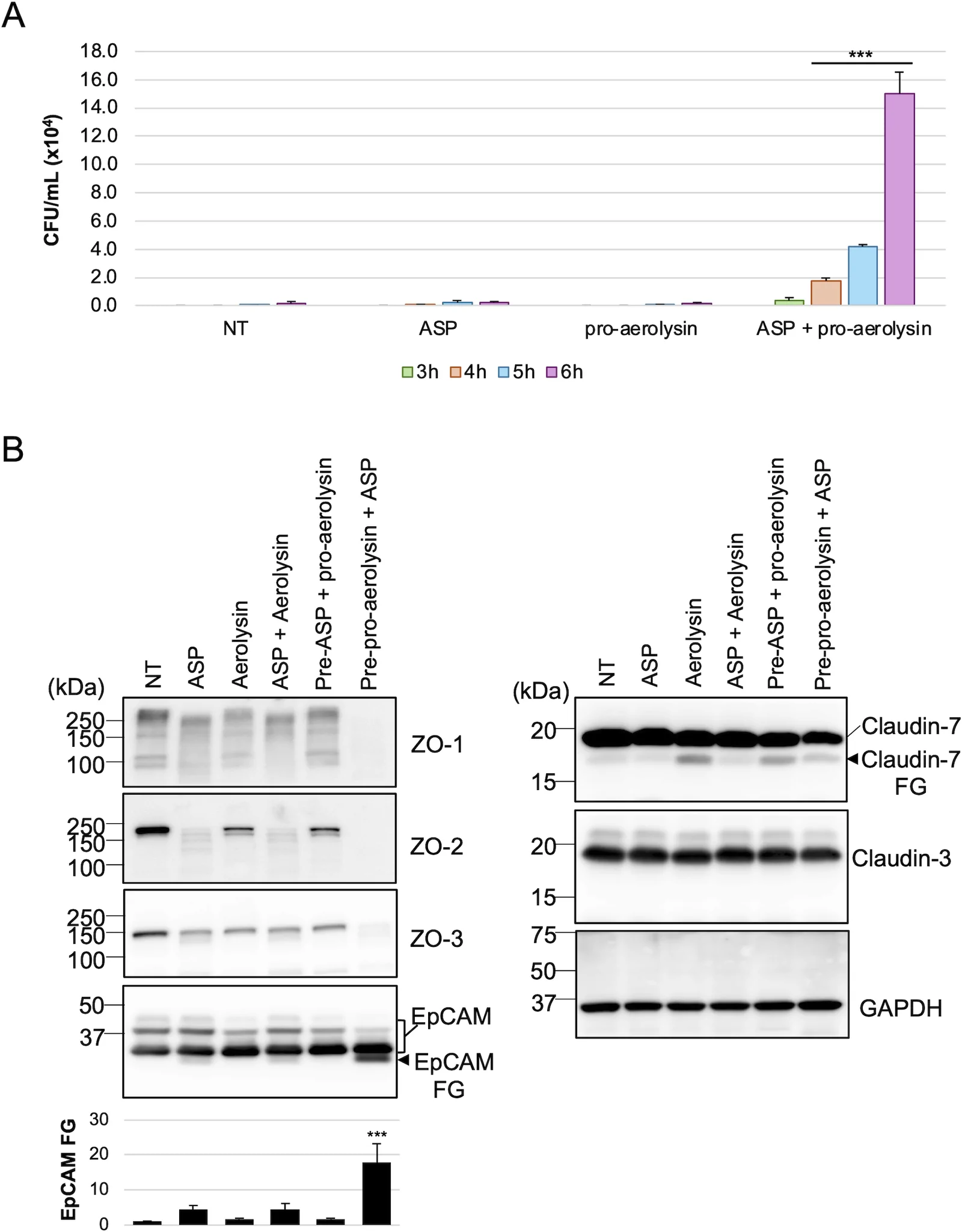
ASP and pro‑aerolysin synergistically enhance bacterial translocation across epithelial monolayers. (**A**) Time-course analysis of bacterial translocation across epithelial monolayers treated with untreated control (NT), ASP alone, pro‑aerolysin alone, or combined ASP + pro‑aerolysin treatment over 6 h. *A. veronii* sobria strain 104 (lacking endogenous ASP and aerolysin production) was used to assess translocation. ASP was used at 100 nM; pro-aerolysin was used at 10 nM. The 104 strain was inoculated at MOI 500. Data represent mean ± SEM (*n* = 3 independent experiments, each performed with an independently prepared T84 monolayer and bacterial inoculum). Statistical analysis was performed using one-way ANOVA, followed by Dunnett’s multiple-comparison test with NT as the control. *P < 0.05, **P < 0.01, and ***P < 0.001. (**B**) ASP-mediated degradation of junctional and adhesion-related proteins. Representative immunoblots showing the effects of different treatments on tight junction proteins (ZO-1, ZO-2, ZO-3, claudin-7, claudin-3), epithelial adhesion molecule (EpCAM), and GAPDH (loading control) at 3 h post-infection. Upper panels show immunoblot results for each protein under six treatment conditions. Lower panel shows quantitative densitometric analysis of EpCAM degradation fragments expressed as fold-change relative to NT (set as 1.0). Data represent mean ± SEM (*n* = 3). Statistical analysis was performed using one-way ANOVA, followed by Dunnett’s multiple-comparison test with NT as the control. *P < 0.05, **P < 0.01, and ***P < 0.001.

To evaluate the direct effects of purified ASP and pro-aerolysin on epithelial barrier function, we quantified bacterial translocation using an *A. veronii* sobria strain 104 (104 strain) that does not produce ASP or aerolysin. This allowed us to assess the effects of exogenously added purified toxins. Bacterial translocation was monitored over a 6-h period under four conditions: untreated control (NT), ASP treatment, pro-aerolysin treatment, and combined ASP + pro-aerolysin treatment (Fig. 5A).

In the untreated control, minimal translocation was observed throughout most of the experiment, with bacterial counts remaining at 0 from 3 to 4 hpi and reaching 1.5 × 10³ ± 1.4 × 10³ CFU/mL by 6 hpi. ASP treatment alone resulted in moderate translocation, with bacterial counts reaching 2.3 × 10³ ± 1.3 × 10³ CFU/mL at 5 hpi and remaining at similar levels (2.0 × 10³ ± 1.3 × 10³ CFU/mL) at 6 hpi. Pro-aerolysin treatment, however, showed delayed and limited translocation, remaining undetectable until 5 hpi when counts reached 4.3 × 10^2^ ± 3.3 × 10^2^ CFU/mL, increasing to 1.3 × 10³ ± 0.8 × 10³ CFU/mL by 6 hpi.

Notably, the combination of ASP and pro-aerolysin produced a dramatic synergistic effect. Translocation began as early as 3 hpi (3.6 × 10³ ± 2.3 × 10³ CFU/mL) and increased exponentially, reaching 1.5 × 10^5^ ± 1.5 × 10^4^ CFU/mL by 6 hpi, far exceeding translocation observed with either ASP or pro-aerolysin alone (Fig. 5A). Statistical analysis using one-way ANOVA, followed by Dunnett’s test, revealed significant differences between ASP + pro-aerolysin and NT at 4, 5, and 6 hpi (*P <* 0.001).

TEER measurements revealed that pro-aerolysin (10 nM) alone caused a rapid and severe decline in epithelial barrier function, decreasing to 49.8% at 1 hpi and reaching approximately 11% by 5 hpi. ASP treatment (100 nM) showed a more gradual decline, decreasing to 71.5% by 5 hpi. The untreated control remained stable at approximately 92%, and the combination of ASP and pro-aerolysin resulted in barrier disruption similar to that observed with pro-aerolysin alone (Fig. S1). Importantly, this analysis revealed a dissociation between TEER and physical bacterial translocation: while pro-aerolysin alone dramatically reduced TEER, it permitted only limited bacterial passage relative to the combined treatment.

Under matching conditions, immunoblotting was performed to examine the effects of ASP and pro-aerolysin on the degradation of junctional proteins (Fig. 5B). Treatment with purified ASP alone resulted in the degradation of ZO proteins, while pro-aerolysin treatment alone induced claudin-7 degradation but not ZO protein degradation.

To investigate potential sequential effects, cells were pretreated with either ASP or pro-aerolysin for 30 min, washed with PBS, and then treated with the alternative toxin. Densitometric quantification of EpCAM degradation fragments expressed as fold-change relative to untreated controls (NT = 1.0) revealed the following: ASP treatment alone resulted in a 4.4-fold increase; pro-aerolysin alone showed a 1.6-fold increase; and simultaneous ASP and pro-aerolysin treatment resulted in a 4.3-fold increase. Pre-treatment with ASP, followed by pro-aerolysin, showed a 1.7-fold increase, whereas pretreatment with pro-aerolysin, followed by ASP, resulted in the most pronounced effect, with a 17.7-fold increase (*P <* 0.01) compared to untreated controls. These quantitative results demonstrated that cells pretreated with pro-aerolysin, followed by ASP, exhibited enhanced degradation of ZO proteins, claudin-7, and EpCAM compared to either treatment alone.

To further investigate the mechanism by which aerolysin contributes to barrier disruption, we examined its effect on the actin cytoskeleton, which is critical for maintaining junctional integrity. T84 cells were treated with pro-aerolysin (100 nM) for 3 h, fixed, and stained with Alexa Fluor 488-conjugated phalloidin to visualize F-actin. Confocal laser scanning microscopy revealed that pro-aerolysin treatment caused a dramatic disruption of the cortical actin cytoskeleton (Fig. S2). In untreated control cells, F-actin was organized in well-defined cortical bundles at cell‒cell junctions and in stress fibers spanning the cell body. In contrast, pro-aerolysin-treated cells exhibited a marked loss of organized actin filaments, with diffuse cytoplasmic fluorescence indicating F-actin depolymerization. This cytoskeletal disruption likely destabilizes junctional protein complexes and enhances susceptibility to ASP-mediated proteolysis.

**Fig 6:**
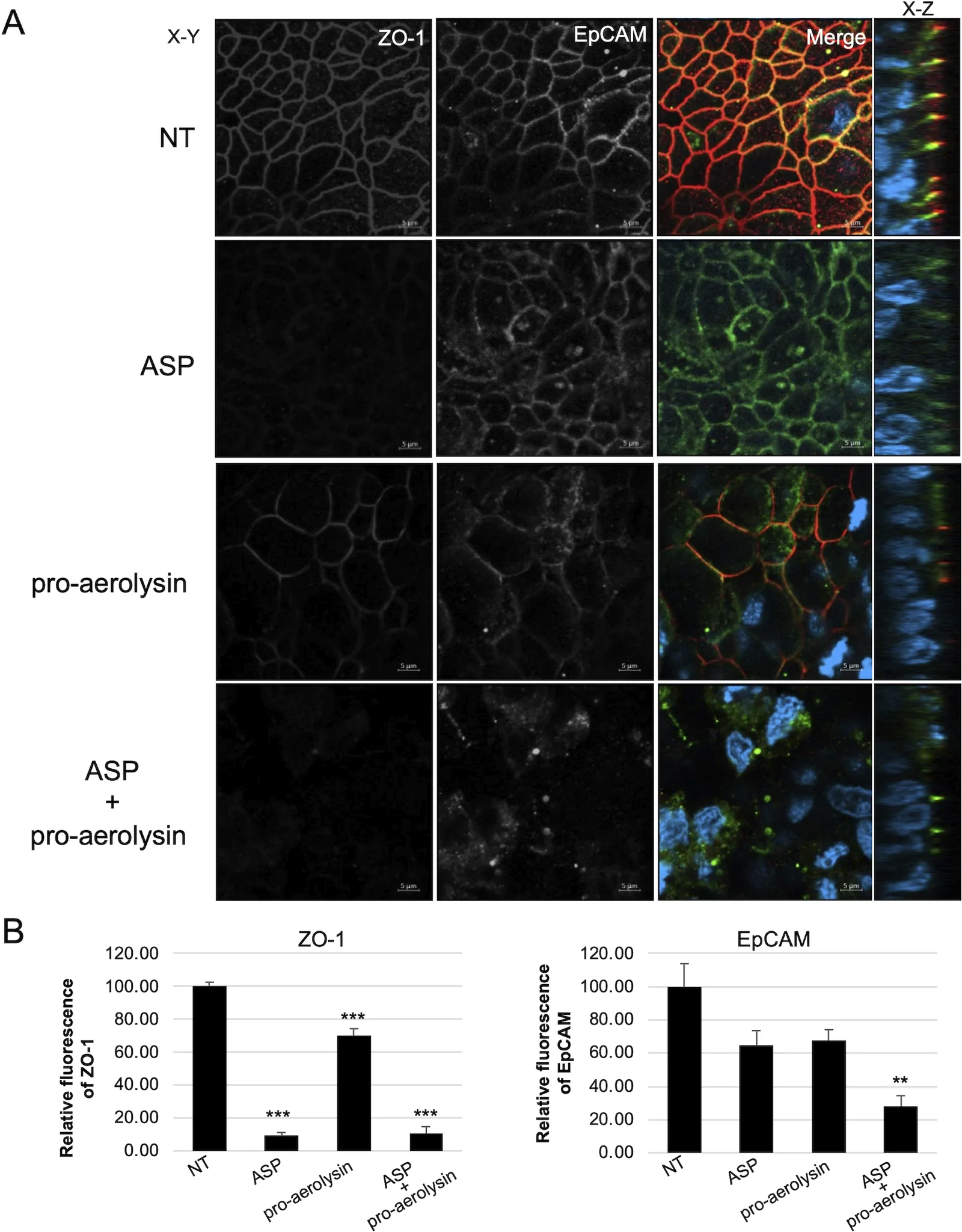
Effects of treatments on epithelial junction proteins. (**A**) Confocal images of ZO-1 (green), EpCAM (red), and merged channels in T84 cells after NT, ASP, pro‑aerolysin, and ASP + pro‑aerolysin treatments. ASP was used at 100 nM; pro-aerolysin was used at 10 nM. X‒Y and X‒Z planes are shown. Scale bar = 5 μm. (**B**) ZO-1 fluorescence intensity quantification (left panel). EpCAM fluorescence intensity quantification (right panel). Fluorescence intensity was quantified from confocal images using Fiji/ImageJ. Data represent mean ± SD (*n* = 3 independent experiments; 5–10 fields per condition per experiment; ≥50 ROIs per condition per experiment). Statistical analysis was performed using one-way ANOVA, followed by Dunnett’s multiple-comparison test with NT as the control. *P < 0.05, **P < 0.01, and ***P < 0.001.

Confocal microscopy was performed to examine the effects of ASP and pro-aerolysin treatments on ZO-1 and EpCAM localization and expression levels (Fig. 6). Under the treatment conditions used for immunofluorescence analysis (3 h), cells remained attached and did not show overt signs of cell death or detachment, ensuring that the observed decreases in fluorescence intensity reflect protein degradation and/or redistribution rather than cell loss. Fluorescence intensity quantification, relative to untreated control cells (set at 100%), revealed significant changes. Specifically, ASP treatment alone reduced ZO-1 fluorescence intensity to 9.7% of control levels, with a marked reduction in ZO-1 continuity at cell‒cell junctions. Pro-aerolysin treatment alone showed a moderate reduction to 70.1% of control levels. The combined ASP and pro-aerolysin treatment resulted in the most severe reduction, decreasing ZO-1 fluorescence to 10.7% of the control level. Statistical analysis revealed significant differences between the ASP and pro-aerolysin treatments (*P <* 0.001) and between the combined ASP and pro-aerolysin treatments and the pro-aerolysin-alone treatment (*P <* 0.001) (Fig. 6B, left panel).

For EpCAM, ASP treatment alone reduced the fluorescence intensity to 64.9% of the control level, whereas pro-aerolysin alone showed a similar reduction to 67.8%. Notably, the combined ASP and pro-aerolysin treatment produced the greatest reduction in EpCAM fluorescence, decreasing it to 28.1% of the control level. Statistical analysis showed significant differences between combined ASP and pro-aerolysin versus ASP alone (*P <* 0.001) and between combined ASP and pro-aerolysin versus pro-aerolysin alone (*P <* 0.001) (Fig. 6B, right panel). The merged images demonstrate that ZO-1 and EpCAM signals co-localize at cell–cell junctions in untreated cells, and that both signals are coordinately diminished upon combined ASP and pro-aerolysin treatment.

### ASP-mediated cleavage of EpCAM

**Fig 7:**
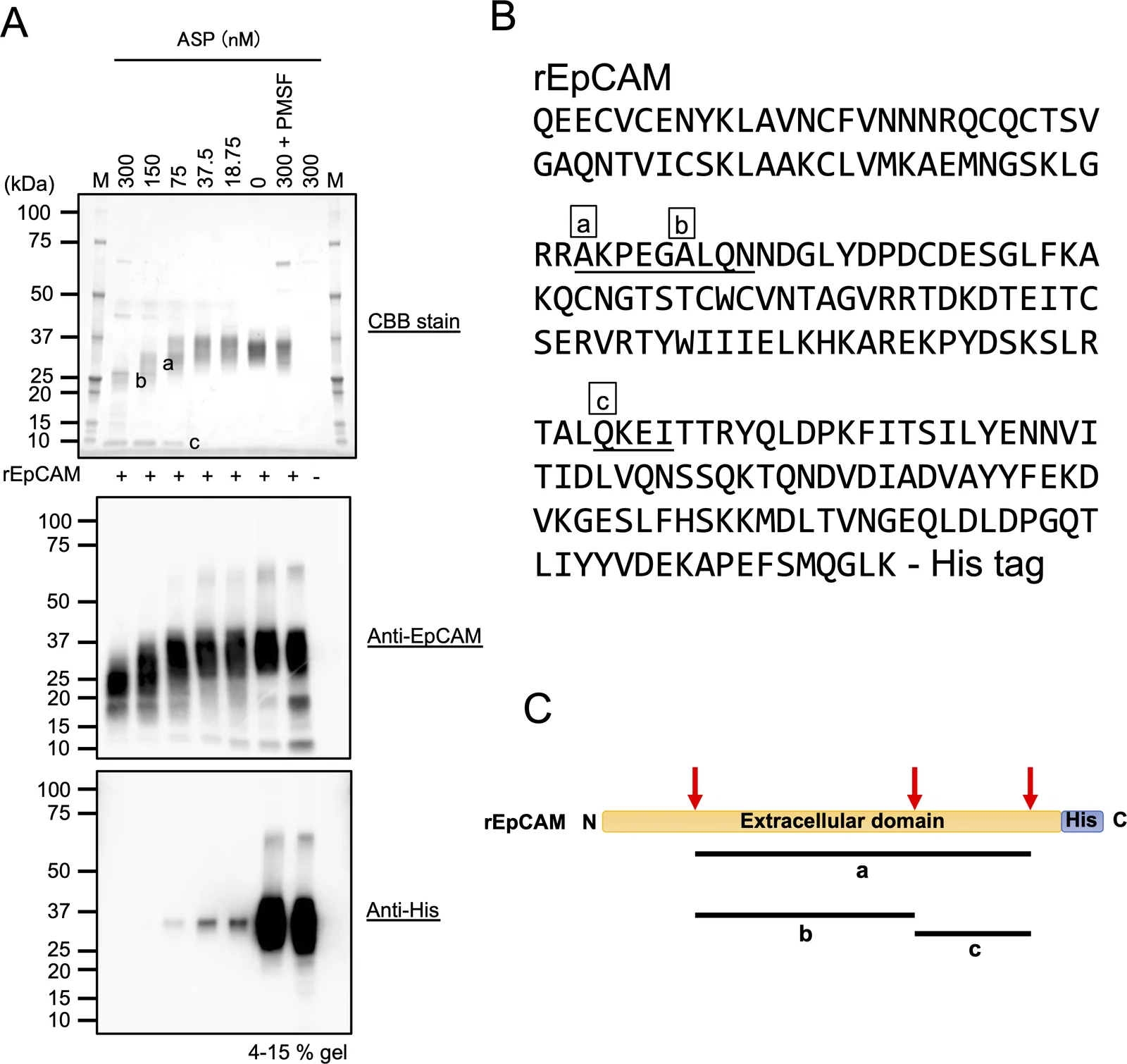
ASP cleaves EpCAM at specific sites within the extracellular domain. (**A**) His-tagged recombinant EpCAM at a final concentration of 0.15 mg/mL was incubated with increasing concentrations of purified ASP for 3 h. Samples were analyzed by SDS-PAGE and visualized by CBB staining (upper panel), anti-EpCAM immunoblotting (middle panel), and anti-His-tag immunoblotting (lower panel). ASP induced concentration-dependent fragmentation of EpCAM. (**B**) N-terminal sequencing of ASP-generated fragments a, b, and c yielded the sequences AKPEG, ALQN, and QKEI, respectively. These represent the major resolvable fragments; additional minor cleavage products were observed but could not be isolated for sequencing. (**C**) Schematic diagram showing the identified ASP cleavage sites (arrows) on the EpCAM extracellular domain. The diagram represents a simplified view of the major cleavage events.

To test whether ASP cleaves the extracellular domain of EpCAM, His-tagged recombinant EpCAM was incubated with various concentrations of purified ASP for 3 h, and the samples were analyzed by SDS-PAGE (Fig. 7A). Electrophoresis revealed ASP concentration-dependent degradation of EpCAM, with progressive fragmentation at higher ASP concentrations. Identical band patterns were observed with both CBB staining and EpCAM antibody detection, confirming the concentration-dependent band shifts. His-tag antibody detection showed a substantial reduction in the signal, even at low ASP concentrations, suggesting that ASP cleaves near the His-tag region. These results indicate that ASP can directly cleave EpCAM.

To determine the specific cleavage sites targeted by ASP, N-terminal sequencing was performed on the ASP-generated fragments designated a, b, and c (Fig. 7B). The N-terminal sequences obtained from fragments a, b, and c were AKPEG, ALQN, and QKEI, respectively. Comparison of these sequences with the EpCAM sequence revealed specific cleavage sites within the extracellular region of EpCAM, as indicated by arrows (Fig. 7C). The three N-terminal sequences identified (AKPEG, ALQN, QKEI) correspond to the major resolvable fragments; additional minor fragments were observed but could not be isolated for sequencing, suggesting that ASP cleaves EpCAM at multiple sites beyond the three identified here.

## DISCUSSION

This study reveals a two-component virulence strategy by which *A. veronii* sobria disrupts the intestinal epithelial barrier through the coordinated actions of ASP and aerolysin. The key findings are that (i) ASP is required for rapid barrier collapse during infection but is not sufficient on its own to recapitulate the phenotype observed during wild-type infection, (ii) ASP can activate pro-aerolysin by proteolytic processing, and (iii) aerolysin and ASP act synergistically—most prominently in an order-dependent manner—to promote extensive junctional and cytoskeletal disruption that enables bacterial translocation. Together, these results provide a mechanistic explanation for how *A. veronii* sobria can breach epithelial defenses and facilitate dissemination.

The coordinated activation of pro-aerolysin by ASP parallels well-characterized precedents in other gram-negative pathogens. For example, in *Vibrio cholerae* El Tor, the pro-hemolysin (HlyA/VCC) is secreted as an inactive precursor (~79 kDa) and requires extracellular proteolytic cleavage by the hemagglutinin/protease (HAP) or host proteases for activation (44). Similar protease-dependent toxin maturation mechanisms have been described in other pathogenic bacteria, underscoring the generality of this virulence strategy.

During infection, ASP deficiency delayed barrier function loss, indicating that ASP is a critical determinant of early disruption. However, purified ASP alone did not recapitulate the rapid barrier collapse despite inducing degradation of ZO-1/2/3 and EpCAM, suggesting that additional bacterial factor(s) amplify the consequences of ASP activity. A major mechanistic insight is the direct cleavage of EpCAM by ASP at multiple extracellular sites. EpCAM contributes to barrier maintenance through its relationship with claudin-7 (19, 45–52), and EpCAM proteolysis can mobilize complexed claudin-7 as part of a physiological repair mechanism (19). Our results suggest that this pathway becomes a vulnerability during infection: ASP-mediated EpCAM cleavage coincides with ZO protein degradation, destabilizing the scaffolding for tight junction assembly, so that EpCAM cleavage becomes barrier-compromising rather than barrier-repairing. Direct visualization of claudin-7 redistribution during toxin exposure will be required to establish this relationship precisely.

Beyond junctional proteolysis, ASP activates pro-aerolysin, linking protease activity to toxin maturation. Our data show that ASP can mediate this activation in place of exogenous proteases, potentially facilitating toxin activity where host protease availability is limited. Importantly, junctional protein cleavage and aerolysin activation are mechanistically separable processes that converge on barrier failure, suggesting that interfering with ASP activity could simultaneously suppress both functions. *In vivo*, body fluids contain abundant protease inhibitors; however, ASP may partially resist such inhibition. Previous work demonstrated that α2-macroglobulin inhibits ASP, but a characteristic “tail nick” in the ASP polypeptide retards this inhibition, allowing prolonged enzymatic activity in plasma (35, 37). Moreover, at the intestinal mucosal surface, where *Aeromonas* infection initiates, the concentration of serum-derived protease inhibitors is expected to be substantially lower than in the bloodstream, potentially providing a permissive environment for ASP activity during early infection.

ASP and aerolysin act synergistically to promote bacterial translocation, with combined treatment achieving significantly higher translocation levels than either toxin alone.

Epithelial barrier failure involves two distinct modes: ionic leakage (TEER reduction) and physical disruption (bacterial passage). While pro-aerolysin alone reduces TEER, the combination with ASP creates structural discontinuities that enable massive bacterial translocation.

Sequential exposure experiments revealed order-dependent synergy: pro-aerolysin pretreatment enhances ASP-mediated protein degradation, likely through membrane permeabilization and cytoskeletal disruption that increases substrate accessibility.

In support of this, aerolysin disrupted cortical F-actin organization, likely destabilizing junctional complexes and increasing substrate accessibility for ASP. Our immunoblotting and functional analyses confirmed that the two toxins exert distinct effects: ASP predominantly degrades ZO proteins and EpCAM, while aerolysin targets claudin-7 and disrupts the actin cytoskeleton. These complementary activities account for their synergistic barrier destruction.

Complementing the above findings, our transposon screen revealed that hemolytic activity is regulated not solely by the presence of toxin genes but also by multiple factors that control aerolysin production. Among the other transposon mutants identified, mutant No. 2 (phosphatidylcholine-sterol acyltransferase) may affect membrane composition and toxin–membrane interactions. Mutant No. 6 (ligand-gated channel protein) could influence ion homeostasis relevant to epithelial barrier function. Mutant No. 7 carrying insertions in genes for 4-hydroxyphenylpyruvate dioxygenase and flagellar hook-associated protein 3 may reflect contributions of metabolic capacity and motility to the full barrier-disrupting phenotype. Further characterization of these mutants may reveal additional layers of regulation within the ASP/aerolysin virulence pathway. In addition to the aerolysin locus, we identified insertions in a tapD homolog (prepilin leader peptidase) and an exeL homolog (T2SS component). Disruption of either gene led to near-complete loss of hemolytic activity, indicating that aerolysin-dependent cytolysis requires intact secretion machinery. Direct measurement of ASP activity confirmed that both the T2SS-deficient (No. 3) and prepilin peptidase-deficient (No. 5) mutants completely lacked extracellular ASP (Fig. S3), demonstrating that T2SS disruption simultaneously abolishes delivery of both toxins. Disruption of lon resulted in a moderate decrease, consistent with a role in protein quality control, though this requires careful interpretation given the pleiotropic nature of Lon. Our findings significantly extend previous research by demonstrating that *Aeromonas* aerolysins are secreted into the extracellular space via the T2SS (41). Notably, loss of T2SS function markedly reduces hemolytic activity, highlighting that secretion defects can have a predominant impact on outcomes, even when toxin genes are present.

Taken together, the data suggest a sequential and cooperative mechanism wherein ASP facilitates both direct junctional proteolysis and aerolysin activation. Concurrently, aerolysin disrupts the plasma membrane and the actin cytoskeleton, compromising the barrier function dependent on claudin-7. Collectively, these processes transition epithelial barrier dysfunction from a state detectable by changes in TEER to a physical breach that permits bacterial translocation. This integrated model provides a mechanistic explanation for the rapid progression from localized mucosal infection to invasive disease.

From a therapeutic standpoint, the dual function of ASP suggests that its inhibition could simultaneously block both junctional proteolysis and toxin activation. Structural insights into ASP (31, 32) could guide the design of specific inhibitors, and combining ASP-targeted approaches with strategies that block aerolysin pore formation may prove more efficacious than targeting either factor alone.

However, this study has several limitations. First, the mechanism by which pro-aerolysin pretreatment enhances susceptibility to ASP-mediated cleavage remains unresolved. Future research should directly investigate whether aerolysin facilitates ASP entry or access to substrates that are normally shielded and should quantify toxin-induced signaling alterations that may affect the conformation or turnover of junctional proteins. Live-cell imaging of junctional remodeling, paired with measurements of intracellular ion fluctuations and cytoskeletal dynamics, would aid in establishing causal relationships between aerolysin exposure and the amplified effects of ASP.

Second, all findings were primarily derived from an *in vitro* epithelial monolayer system. *In vivo*, epithelial barriers are subject to influences from mucus, immune responses, microbiota, and host proteases, which may modify toxin availability and epithelial responses. Validation in more physiologically relevant models, such as intestinal organoids and animal infection models, is crucial to ascertain the contribution of the ASP–aerolysin pathway to dissemination and disease severity.

Third, it remains unclear whether similar cooperative mechanisms function across other clinically significant *Aeromonas* species. Comparative functional studies across species and strains with distinct virulence factor repertoires will be necessary to establish the general applicability of the two-component strategy described in this report. Once the epithelial barrier is breached, *Aeromonas* would encounter the subepithelial immune system, including tissue-resident macrophages, dendritic cells, and neutrophils, as well as complement and other humoral defense mechanisms. The ability of ASP to degrade complement components (C5) (34) and to activate prothrombin (33), combined with aerolysin-mediated cytotoxicity toward immune cells, may enable the bacterium to resist immediate immune clearance and establish systemic infection. Indeed, recent work has demonstrated that aerolysin-producing *Aeromonas* variants can deplete tissue-resident macrophages in the colon, impairing this innate immune barrier and promoting susceptibility to colitis (43). However, the precise interplay between *Aeromonas* virulence factors and the host immune response following epithelial translocation remains to be elucidated *in vivo*.

In conclusion, this study elucidates a coordinated two-component mechanism by which *A. veronii* sobria disrupts the epithelial barrier. ASP functions both in direct cleavage of junctional and adhesion proteins and in the activation of pro-aerolysin. Aerolysin-induced membrane and cytoskeletal perturbations further enhance ASP-mediated effects. The synergy between these factors, including the observed order dependence, provides a mechanistic basis for efficient epithelial breach and bacterial translocation. These findings offer a framework for understanding invasive *A. veronii* sobria infections and support the development of targeted anti-virulence strategies focused on the ASP–aerolysin pathway.

## MATERIALS AND METHODS

### Human intestinal epithelial cell culture

Cell culture was performed as described previously (38). Briefly, the intestinal epithelial cell line T84 (ECACC) was grown and maintained at 37˚C in a 5% CO_2_ atmosphere in a 1:1 mixture of DMEM and Ham’s F-12 nutrient mixture supplemented with 10% FBS, 15 mM HEPES, 14.3 mM NaHCO_3_, and antibiotics/antimycotics. For experiments, cells were grown to a confluent monolayer in collagen-coated polycarbonate Transwell inserts (0.33 cm^2^, Corning Life Sciences) or 12-well plates. Cells were used when TEER was between 600 and 1000 Ω·cm^2^, which was 10–14 d post-plating.

### Transepithelial electrical resistance

Transepithelial electrical resistance (TEER) was measured directly in the culture medium using an epithelial volt–ohm meter (Model Millicell-ERS; Millipore, Cambridge, MA). TEER values were calculated by subtracting the background TEER from blank filters, and then multiplying by the surface area of the filter (0.33 cm²). Measurements were performed at the indicated time points. Data are expressed as a percentage of the initial TEER value (time zero) for each insert.

### Bacterial translocation assay

The bacterial translocation assay was carried out as described (39). Briefly, bacterial cells were suspended in Hanks' balanced salt solution (HBSS) containing 15 mM HEPES (pH 7.4) and added to the apical side of T84 cells grown on Transwell filters (pore size 3.0 µm) at an MOI of 500. The medium from the lower chamber was collected every hour for 6 h post-infection (hpi), and bacterial counts were determined by plating on LB agar.

### Bacterial strain culture

*A. veronii* sobria wild-type strain (w288), ASP-deficient strain (Δ*asp*), and strain 104 (which does not produce ASP and aerolysin) were used. Bacterial strains were cultured in LB medium at 37˚C with shaking. After culture, the turbidity of the bacterial suspension was measured, and the suspension was diluted with PBS to achieve OD600 = 0.5‒0.6 for use in experiments.

### Western blot analysis

Western blot analysis was performed as described previously (39), with the addition of EpCAM antibody detection. Briefly, after bacterial infection for 3 h, cells were lysed, and proteins were separated by SDS-PAGE, then transferred to PVDF membranes. Primary antibodies used included ZO-1, ZO-2, ZO-3, claudin-3, claudin-7, GAPDH (as previously described), and EpCAM (ab223582, Abcam) at a dilution of 1:1,000. Densitometric quantification was performed using ImageJ software (NIH). Band intensities were normalized to the internal control GAPDH.

### EpCAM degradation fragment quantification

Densitometric analysis of immunoblot bands was performed using ImageJ software. Band intensities were quantified using ImageJ and normalized to loading controls.

### Immunofluorescence confocal laser scanning microscopy and F-actin visualization

Immunofluorescence staining and confocal microscopy were performed as described previously (38), with the addition of EpCAM detection and F-actin visualization. Briefly, T84 cells grown on Transwell filters were treated with bacterial solutions or toxins, fixed with 4% paraformaldehyde, permeabilized, and stained with primary antibodies, including ZO-1 and EpCAM (ab223582, Abcam) at a dilution of 1:1000. For F-actin visualization, fixed and permeabilized cells were stained with Alexa Fluor 488-conjugated phalloidin (Thermo Fisher Scientific) according to the manufacturer’s instructions. Fluorescence signals were visualized with a confocal laser scanning microscope (LSM800, Carl Zeiss). Fluorescence intensity was quantified as previously described (53). Briefly, regions of interest (ROIs) were segmented using ImageJ, version 1.53c (54), and relative fluorescence intensities of at least 50 ROIs per condition were measured and expressed as mean ± SD. Values were normalized to untreated control cells.

### Construction and analysis of transposon mutants

Transposon mutagenesis was performed as described (55). Briefly, a mariner-based transposon delivery plasmid, pSC189 (56), was introduced into *A. veronii* sobria strain 288 cells by plate mating from *E. coli* SM10λ*pir* (57) at 20˚C. The mating was plated on LB agar containing kanamycin, and transposon-inserted kanamycin-resistant mutants were selected. A total of 5,472 transposon insertion mutants were screened for reduced epithelial cell detachment activity by visual assessment under microscopic observation. Seven mutants showing reduced activity were selected for further analysis, and cell extracts from infected intestinal epithelial cells were prepared to detect ZO-1 and ZO-2 by western blotting. For identification of transposon insertion sites, genomic DNA was isolated from selected mutants, digested with SphI, and then self-ligated using T4 DNA ligase. Competent *E. coli* DH5α(λ*pir*) cells were transformed with the ligation reaction mixture and plated on LB agar containing kanamycin. The position of transposon insertion was determined by sequencing the transposon‒genome junction using the MAREC6 primer (5′-GCTTGTCATCGTCATCCTTGTAATCG-3′) (58).

### Measurement of hemolytic activity

Culture supernatants from each mutant strain were collected, and hemolysis assays were performed using sheep red blood cells (SRBC). Culture supernatants and 2% SRBC suspension were mixed 1:1 and incubated at 37˚C for 1 h, followed by centrifugation (1,000 × *g*, 5 min). The absorbance (OD_540_) of the supernatant was measured to evaluate hemolytic activity.

### Measurement of ASP protease activity

ASP proteolytic activity was measured using the synthetic peptide substrate Boc-Glu-Lys-Lys-MCA (Peptide Institute, Osaka, Japan) as described previously (31). Culture supernatants from transposon mutant strains were incubated with the substrate at 37°C for 1 h, and proteolytic activity was determined by measuring fluorescence intensity (excitation 340 nm, emission 440 nm) to monitor the release of the fluorescent product MCA (7-amino-4-methylcoumarin).

### Purification of aerolysin precursor

The aerolysin gene was cloned into the pET-11 vector to produce recombinant protein with both His-tag and GST-tag and expressed in *E. coli* BL21(DE3) strain. Expression was induced with IPTG (final concentration of 0.2 mM), and cells were cultured at 16˚C for 20 h before harvesting. Bacterial cells were disrupted by sonication, and the lysate was first purified using a HisTrap HP column (Cytiva Life Sciences). The eluted fractions were then applied to a Glutathione Sepharose 4B column (Cytiva Life Sciences), and PreScission Protease (Cytiva Life Sciences) was added for tag removal by incubation at 4˚C overnight. The eluted solution after protease treatment was used as the purified pro-aerolysin solution.

### Protease treatment and hemolytic activity measurement

Purified pro-aerolysin (100 nM) was treated with trypsin (100 nM) or purified ASP (100 nM) and incubated at 37˚C for 30 min. After the reaction, hemolytic activity was measured using the method described above.

### Analysis of protein degradation patterns and cleavage sites

Samples after protease treatment were subjected to SDS-PAGE (10% polyacrylamide gel) electrophoresis, and bands were detected by Coomassie Brilliant Blue staining. Target bands were excised and transferred to PVDF membranes. N-terminal amino acid sequences were analyzed by Edman degradation using a peptide sequencer (PPSQ-31A, Shimadzu) to identify cleavage sites.

### Recombinant EpCAM cleavage assay

Recombinant His-tagged human EpCAM protein was purchased from Abcam (ab151338). His-tagged recombinant EpCAM at a final concentration of 0.15 mg/mL was incubated with various concentrations of purified ASP at 37˚C for 3 h, and samples were analyzed by SDS-PAGE and immunoblotting as described above.

### Statistical analysis

All experiments were repeated independently at least three times. Data are presented as mean ± SEM (or mean ± SD, where indicated). For comparisons among multiple groups, statistical significance was assessed by one-way analysis of variance (ANOVA), followed by Dunnett’s multiple comparisons test using EZR, an R-based interface. For time-course experiments, statistical comparisons were performed at each time point using one-way ANOVA, followed by Dunnett’s test. *P* values < 0.05 were considered statistically significant.

## Data Availability

All data supporting the findings of this study are available within the article and its supplemental materials. Additional data are available from the corresponding author upon reasonable request.

## SUPPLEMENTAL MATERIAL

Fig. S1 jb.00141-26-s0001.tiff TEER time course in T84 monolayers treated with pro‑aerolysin, ASP, or their combination.

Fig. S2 jb.00141-26-s0002.tiff Disruption of the actin cytoskeleton by pro‑aerolysin visualized by phalloidin staining and confocal microscopy.

Fig. S3 jb.00141-26-s0003.tiff ASP protease activity in transposon mutant culture supernatants. Fluorescence intensity was measured using Boc-Glu-Lys-Lys-MCA substrate.

Supplemental figures legends jb.00141-26-s0004.docx Legends for Fig. S1 to S3.

ASM does not own the copyrights to Supplemental Material that may be linked to, or accessed through, an article. The authors have granted ASM a non-exclusive, world-wide license to publish the Supplemental Material files. Please contact the corresponding author directly for reuse.
