# Supplementary material for: Aeromonas veronii biotype sobria serine protease activates aerolysin and cleaves EpCAM to breach the epithelial barrier
Source: J Bacteriol. 2026 Jul 10;208(8):e00141-26. doi: 10.1128/jb.00141-26 (PMC13492141; doi:10.1128/jb.00141-26)
Supplement: Supplementary file 1 [file jb.00141-26-s0004.docx]

**SUPPLEMENTAL MATERIAL**

**Figure S1 Pro-aerolysin induces rapid TEER decline independent of ASP.**

Time-course of TEER changes in T84 cell monolayers treated with pro‑aerolysin (10 nM), ASP (100 nM), or their combination. Pro-aerolysin alone caused rapid barrier disruption (49.8% at 1 h, ~11% at 5 h), while ASP alone showed gradual decline (71.5% at 5 h). The combination treatment exhibited similar kinetics to pro‑aerolysin alone. Control monolayers maintained ~92% TEER. Data represent mean ± SEM (n = 3). Despite dramatic TEER reduction, pro‑aerolysin alone permitted limited bacterial translocation compared to combined treatment.

**Figure S2 Aerolysin disrupts the actin cytoskeleton in intestinal epithelial cells.**

Confocal laser scanning microscopy images of T84 cells stained with Alexa Fluor 488-conjugated phalloidin to visualize F-actin. Left panel: Untreated control cells showing well-organized cortical actin bundles at cell‒cell junctions (arrows) and stress fibers. Right panel: Cells treated with pro‑aerolysin (100 nM) for 3 h showing marked loss of organized actin filaments and diffuse cytoplasmic fluorescence, indicating F-actin depolymerization. Scale bar = 5 µm. Representative images from three independent experiments are shown.

**Figure S3 ASP substrate cleavage activity in transposon mutant supernatants.**

Culture supernatants were incubated with Boc-Glu-Lys-Lys-MCA substrate at 37°C for 1 h, and fluorescence intensity was measured. Data represent mean ± SEM (n = 3).
